# Bladder metastases of appendiceal mucinous adenocarcinoma: a case presentation

**DOI:** 10.1186/1471-2407-10-62

**Published:** 2010-02-23

**Authors:** Gianluigi Taverna, Matteo Corinti, Piergiuseppe Colombo, Fabio Grizzi, Mauro Severo, Alessando Piccinelli, Guido Giusti, Alessio Benetti, Paolo A Zucali, Pierpaolo Graziotti

**Affiliations:** 1 Department of Urology, Via Manzoni 56 - 20089 Rozzano, Milan, Italy; 2 Department of Pathology, Via Manzoni 56 - 20089 Rozzano, Milan, Italy; 3 Laboratories of Quantitative Medicine, Via Manzoni 56 - 20089 Rozzano, Milan, Italy; 4 Department of Medical Oncology, IRCCS Istituto Clinico Humanitas, Via Manzoni 56 - 20089 Rozzano, Milan, Italy

## Abstract

**Background:**

Appendiceal adenocarcinoma is rare with a frequency of 0.08% of all surgically removed appendices. Few cases of appendiceal carcinoma infiltrating the bladder wall for spatial contiguity have been documented.

**Case Presentation:**

A case is reported of a 45-years old woman with mucinous cystadenocarcinoma of the appendix with bladder metastasis. Although ultrasonography and voided urinary cytology were negative, abdomen computed tomography (CT) scan and cystoscopy and subsequent pathological examination revealed a mass exclusively located in the anterior wall of the bladder. Histopathology of the transurethral bladder resection revealed a bladder adenocarcinoma [6 cm (at the maximum diameter) × 2,5 cm; approximate weight: 10 gr] with focal mucinous aspects penetrating the muscle and perivisceral fat. Laparotomy evidenced the presence of a solid mass of the appendix (2,5 cm × 3 cm × 2 cm) extending to the loco-regional lymph nodes. Appendectomy and right hemicolectomy, linfoadenectomy and partial cystectomy were performed. The subsequent pathological examination revealed a mucinous cystadenocarcinoma of the appendix with metastatic cells colonising the anterior bladder wall and several colic lymph nodes.

**Conclusions:**

The rarity of the appendiceal carcinoma invading the urinary bladder and its usual involvement of nearest organs and the posterior bladder wall, led us to describe this case which demonstrates the ability of the appendiceal cancer to metastasize different regions of urinary bladder.

## Background

The appendiceal adenocarcinoma invading the urinary bladder is extremely rare [1-4], and it is very difficult to be diagnosed before the surgical inspection. Here we describes a case of appendiceal cystoadenocarcinoma metastising the bladder anterior wall diagnosed in a patient with monosymptomatic episodes of gross hematuria. Our diagnostic schema and the adopted pharmacological treatment are also discussed.

## Case Presentation

**Figure 1:**
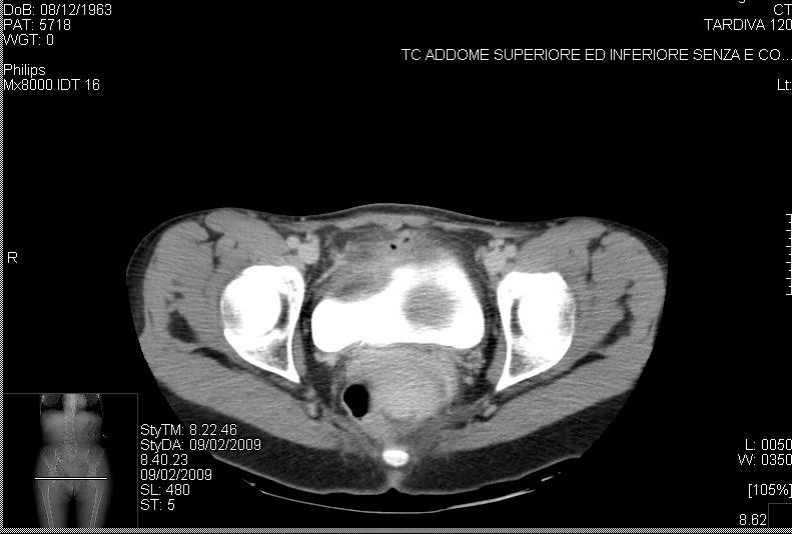
**Enhanced abdomen CT scan demonstrate a 6 cm × 2,5 cm anterior bladder mass**.

**Figure 2:**
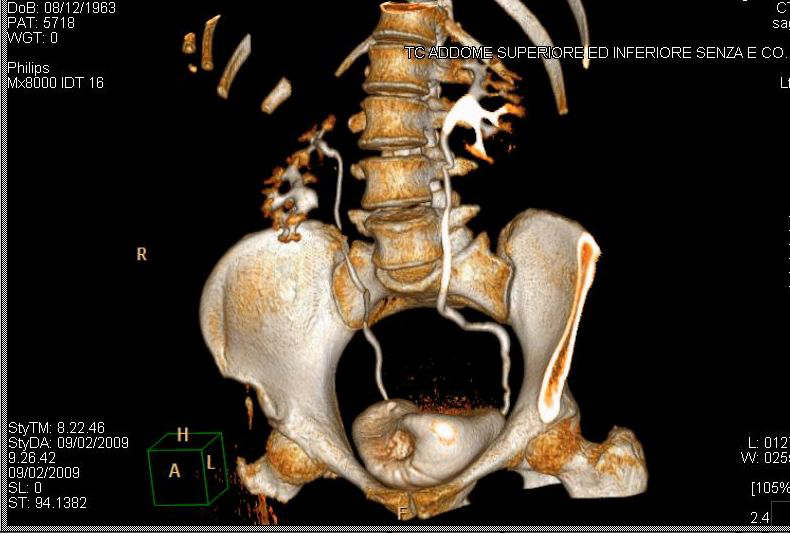
**Enhanced abdomen CT scan reconstruction showing the bladder lesion at the level of the anterior wall**.

**Figure 3:**
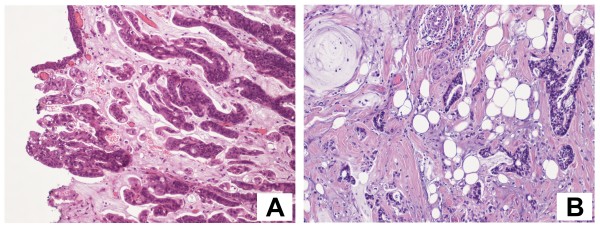
**The first pathological examination revealed a mucinous adenocarcinoma suspicious for primary of the bladder (A), infiltrating the full thickness of the visceral wall (B)**. Objective magnification: 20×.

**Figure 4:**
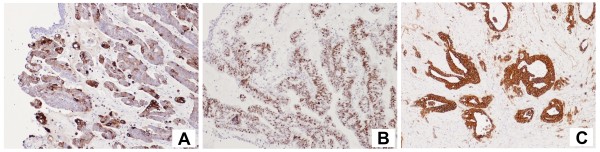
**Tumoural cells were found immunopositive for cytokeratin 7 (A), CDX2 (B), and β-catenin (C)**. Objective magnification: 20×.

**Figure 5:**
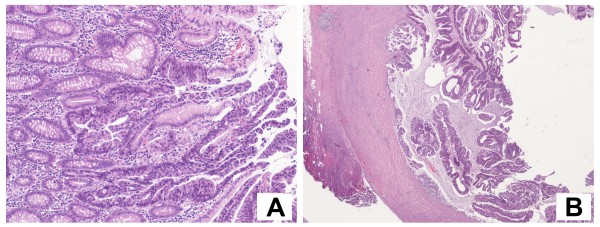
**The definitive pathological examination revealed a mucinous cystoadenocarcinoma of the appendix invading the caecal wall with metastasis of the anterior wall of the bladder (A)**. Adjacent to the tumour in the intestinal mucosa foci of high grade dysplasia/adenocarcinoma in situ were also identified thus confirming the appendiceal origin of the primary tumour (B). Objective magnification: 20×.

A 45-years old woman presented at our health centre (IRCCS Istituto Clinico Humanitas, Rozzano, Milan, Italy) with some episodes of gross hematuria. The ultrasonography and voided urinary cytology, and the physical examination were negative. The patient never had any gastrointestinal symptom and presented a negative regress medical history. The enhanced abdomen computed tomography (CT) scan evidenced a solid mass [6 cm (at the maximum diameter) × 2,5 cm] at the level of the anterior bladder wall (Figures 1 and 2). Cystoscopy confirmed the presence of a 6 cm extended solid broad-based mass localized at the level of the anterior wall of the bladder. A transurethral resection was therefore performed and the first pathological examination revealed a mucinous adenocarcinoma suspicious for primary of the bladder (approximate weight: 10 gr), infiltrating the full thickness of the visceral wall (Figures 3A and 3B). Tumoural cells were found immunopositive for cytokeratin 7, CDX2 (nuclear immunoreactivity), and β-catenin (membranous immunoreactivity) (Figures 4A-C). Subsequent laparotomy showed a thickened appendix and caecum adherent to the bladder posterior wall without its infiltration. Intraoperative frozen analysis revealed foci of adenocarcinoma with mucinous components suspicious of colonic origin in sections of appendiceal tissue, and the presence of tumoral cells colonizing a mesenteric lymph-node. Appendectomy and right hemicolectomy with ileocolic anastomosis, lymphadenectomy and partial cystectomy limited to the anterior wall of the bladder was therefore performed. The definitive pathological examination revealed a mucinous cystoadenocarcinoma of the appendix (2,5 cm × 3 cm × 2 cm) invading the caecal wall with metastasis of the anterior wall of the bladder (Figure 5A). Adjacent to the tumour in the intestinal mucosa foci of high grade dysplasia/adenocarcinoma *in situ *were also identified (Figure 5B). In addition, metastasis were found in 15/37 (40%) peri-colic lymph-nodes. The pathological staging was: pT4N2M1 (TNM classification), stage D (Dukes, Astler and Coller classification). A mutational analysis for k-Ras was done and the tumour resulted k-Ras mutated. A radiological tumour assessment after surgery showed a suspicion of disease persistence to the intra-abdominal lymph-nodes and to the pelvis (cystic lesion), with an increased of carcinoembryonic antigen (CEA) serum level (29 ng/ml). From May to October 2009 the patient received 12 cycles of a first line chemotherapy with fluorouracil, folic acid, irinotecan (FOLFIRI regiment), and bevacizumab. Radiological tumour assessment after 4 and 8 cycles showed stable disease with a reduction of CEA serum level (14 ng/ml and 10 ng/ml, respectively). The radiological tumour assessment at the end of chemotherapeutic treatment (November 2009) showed a volumetric progressive disease at the level of the pelvic cystic lesion (confirmed by cytological analysis), whereas a further reduction of CEA serum level (7 ng/ml) was observed. The patient is going to receive a second line chemotherapy with fluorouracil, folic acid (de GRAMONT regiment), and bevacizumab. At this time no further bladder lesions have been detected during a cystoscopy inspection.

## Conclusions

Appendiceal adenocarcinoma is rare with a frequency of 0.08% of all surgically removed appendices. To our best knowledge it was reported few cases of appendiceal carcinoma infiltrating the bladder wall for spatial contiguity [1-11]. It is recognized that tumours of the pelvic organs (*i.e. *ovary, colon and rectum) may invade the urinary bladder [10,11]. In these cases symptoms presenting complaints urinary disease and eventually symptoms referable to the gastrointestinal tract [2]. All patients undergo appendectomy, segmental bladder resection or cystectomy and segmental caecal resection or right colectomy [5-9]. In our case the bladder neoplasia was found of metastatic origin and not locally invading from the primary appendiceal tumoural site. The primary lesion of the appendix was nearby the posterior bladder wall without local invasion. Thus confirming that the anterior bladder lesion was a secondary localization of appendiceal carcinoma. Ultrasonography and abdomen enhanced CT scan unrevealed any gastrointestinal primary lesion. We therefore decided for a partial cystectomy, because metastases were found in the anterior portion of the bladder, completely unconnected from the primary appendiceal tumour. We have considered the possibility of a synchronous cancer, but the overlap between the morphologic features of the tumour and the clinical characteristics of the disease strongly support the metastatic nature of the bladder adenocarcinoma. In addition, we found multiple metastasis at the level of colic lymph-nodes and not at the level of bladder lymph-nodes. This supports the fact that the bladder lesion is of metastatic origin and not definable as a primary tumoural lesion. Some explanations of the involved metastatic pathways might linked to the fact that tumoural clones may have embolized lymphatic vessels located under the peritoneal serous membrane of the appendiceal-caecum region, and after the colonization of a high number of loco-regional lymph-nodes, tumoural cells might have reach the peri-bladder lymph-nodes. However, this hypothesis is in contrast with the fact that no new bladder lesions have been found during the follow-up of the patient. In our presented case the anatomical characteristics of non contiguity between the anterior bladder wall and the appendix, caecum and peritoneum, and the different neoplastic lymph-node development between bladder and colic cancer led us to hypothesize that the metastatic pathway was haematogenous.

The rarity of the appendiceal carcinoma invading the urinary bladder and its usual involvement of nearest organs and the posterior bladder wall, led us to first describe this case which demonstrates the ability of the appendiceal carcinoma to metastasise different portions of urinary bladder.

## Competing interests

The authors declare that they have no competing interests.

## Authors' contributions

GT, MC, MS, AB performed all surgical procedures and drafted the manuscript. PC confirmed all histological findings. FG, AP, GG, PAZ, PG helped to draft the manuscript. All authors read and approved the final manuscript.

## Consent

Written informed consent was obtained from the patient for publication of this case report and any accompanying images. A copy of the written consent is available for review by the Editor-in-Chief of this journal.

## Pre-publication history

The pre-publication history for this paper can be accessed here:

http://www.biomedcentral.com/1471-2407/10/62/prepub
